# Signature of Topological Surface Bands in Altermagnetic Weyl Semimetal CrSb

**DOI:** 10.1021/acs.nanolett.5c00482

**Published:** 2025-04-28

**Authors:** Wenlong Lu, Shiyu Feng, Yuzhi Wang, Dong Chen, Zihan Lin, Xin Liang, Siyuan Liu, Wanxiang Feng, Kohei Yamagami, Junwei Liu, Claudia Felser, Quansheng Wu, Junzhang Ma

**Affiliations:** † Department of Physics, City University of Hong Kong, Kowloon, Hong Kong, China; ‡ Institute of Physics and Beijing National Laboratory for Condensed Matter Physics, Chinese Academy of Sciences, Beijing 100190, China; § University of Chinese Academy of Science, Beijing 101408, China; ∥ College of Physics, Qingdao University, Qingdao 266071, China; ⊥ Max Planck Institute for Chemical Physics of Solids, 01187 Dresden, Germany; # Centre for Quantum Physics, Key Laboratory of Advanced Optoelectronic Quantum Architecture and Measurement (MOE), School of Physics, Beijing Institute of Technology, Beijing 100081, China; ◆ Japan Synchrotron Radiation Research Institute, 1-1-1, Sayo-cho, Sayo-gun, Hyogo 679−5198, Japan; ○ Department of Physics, The Hong Kong University of Science and Technology, Hong Kong, China

**Keywords:** g-wave altermagnetism, topological Weyl semimetal, ARPES, first-principles calculation

## Abstract

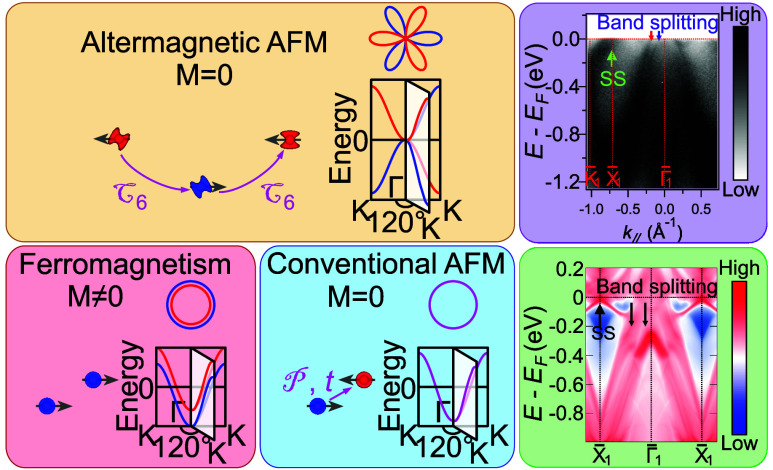

As a special type of collinear antiferromagnetism (AFM), altermagnetism has garnered significant research interest recently. Altermagnets exhibit broken parity-time symmetry and zero net magnetization, leading to substantial band splitting in the momentum space. Meanwhile, parity-time symmetry breaking is a prerequisite for nontrivial band topology in Weyl physics. When there is band crossing, it is usually easy to generate Weyl nodes. Weyl semimetal states have been theoretically proposed in altermagnets; rare reports of experimental observation have been made up to this point. Using angle-resolved photoemission spectroscopy (ARPES) and first-principles calculations, we systematically studied the electronic structure of room-temperature altermagnet candidate CrSb. We clearly observed the band spin splitting and signature of topological surface states on the (100) cleaved side surface close to the Fermi level originating from bulk band topology. Our results imply that CrSb contains interesting nontrivial topological Weyl physics, in addition to being an excellent room temperature altermagnet.

In condensed matter physics, the lifting of electronic band spin degeneracy in crystalline solids can induce novel physical properties and hold promise for electronic device applications.^1,2^ Typically, spin–orbit coupling (SOC) in noncentrosymmetric systems is a key factor driving spin splitting, especially in elements with large atomic numbers.^3^ However, this limits the potential material candidates among elements with small atomic numbers. In recent years, theoretical analysis proposed that collinear antiferromagnetic (AFM) order without parity-time symmetry (PT) and τ*U* symmetry (where τ is a translation operation and *U* is a spin rotation operation) can possess dramatic spin splitting without the need to consider SOC.^4,5^ This increases the range of material candidates for spin splitting by including compounds made up of small atomic number elements. As a result of their distinct magnetic characteristics, collinear AFM materials have started to garner a lot of attention from researchers.^6−13^ Especially interesting is that the intrinsic crystal (*C*-) symmetry ensuring the zero magnetization in AFMs will force contrasting spin polarization at different valleys or momenta paired by this crystal symmetry, forming the *C*-paired spin-valley locking,^11^ which leads to the unique noncollinear spin current generation and piezomagnetism.^9,11,14^ Scientists refer to this kind of AFM arrangement as “altermagnetism”.^15,16^ The unique physical characteristics of altermagnets have been verified by lots of recent theoretical and experimental investigations.^17−23^ The recent advancements in altermagnets include novel physical phenomena associated with altermagnetism.^24,25^ For more discussion about the properties of altermagnetism, please see the Supporting Information.

Meanwhile, the breaking of symmetry usually induces novel topological quantum states. Dirac nodes divide into Weyl nodes when parity-time symmetry is broken, giving rise to exotic physical phenomena such as massless quasiparticles, open surface Fermi arcs, chiral anomaly, and the anomalous Hall effect.^26−33^ Significant spin splitting is required for an ideal Weyl semimetal in order to generate well-separated Weyl nodes.^31−34^ In the majority of perfect topological semimetals, the SOC is essential. Scientists have recently realized that altermagnets present a special chance for enormous spin splitting to drive perfect topology.^35−37^ Experimental observations on the nontrivial topological band structures of altermagnets are still rare, though, as this topic is still in its beginning. Nontrivial topology combined with altermagnetism may give rise to new physical properties combining the features of altermagnetism and topological physics. Altermagnetic Weyl semimetals may find use in spintronic devices as a result of this synergy.

Calculations have predicted plenty of potential altermagnet candidates,^15,16^ and recent experimental research has validated several of them.^20,21,38,39^ Notably, among these candidates, CrSb shows significant large spin splitting up to 1 eV with critical temperature above room temperature.^22^ As previously highlighted, altermagnets hold significant scientific merit and promise for various applications.^15,16,18,19^ However, there is still a scarcity of experimental studies of the electronic topological structures of altermagnets. Electronic bands in CrSb epitaxial thin films were investigated with high photon energy soft X-ray angle-resolved photoemission spectroscopy (ARPES) in recent studies, and the band dispersions indicate an altermagnetic band structure in CrSb.^21^ Nevertheless, the high-resolution band structure, surface states, and topology of CrSb have not yet been thoroughly examined in the low photon-energy region. Recently, four preprints regarding the ARPES investigation have concentrated on the band spin splitting, and one has claimed the topology and surface states on the (001) top surface of CrSb^40−43^ during the preparation of the current manuscript. Meanwhile, (100) side surface states have not yet been reported to date. In this study, using ARPES combined with first-principles calculations, we systematically examined the electronic structure of the room-temperature altermagnet candidate CrSb from the side (100) surface, employing both soft X-ray and vacuum ultraviolet (VUV) ARPES. We observed clear spin splitting in the generic momentum space away from high-symmetry lines. Additionally, we detected surface state bands on the (100) cleaved side surface near the Fermi level, which emerge from bulk topology when compared with theoretical calculations. Our results have identified signs that indicate that CrSb is an altermagnetic Weyl semimetal.

CrSb hosts a NiAs-type structure with space group *P*63/*mmc* (#194),^44^ as shown in Figure 1b, and Figure 1c displays the corresponding 3D Brillouin zone (BZ). Two distinct Sb atoms are interpolated between Cr layers along the *c* axis and encircled by six Cr atoms. A type AFM order with an in-plane FM structure and an out-of-plane AFM structure with two spin sublattices is formed by the magnetic momentum carried by the Cr atoms. Because of nonequivalent Sb atoms by means of translation operations, the magnetic lattice structure cannot be restored by parity-time symmetry. Instead, the two sublattices can be related by the M_*z*_ mirror operation or by the combination of rotation operation C_6*z*_ and translation operation as shown in Figure 1a. The X-ray diffraction results are shown in Figure S1, which agree well with the default data. The broken parity-time symmetry and the special sublattice structure define CrSb as g-wave altermagnetism with large spin splitting, as demonstrated in Figure 1a. The X-ray photoemission spectroscopy (XPS) results are plotted in Figure 1d, with both Cr and Sb elements clearly resolved. The photograph of CrSb samples is shown in Figure 1e for reference.

**Figure 1:**
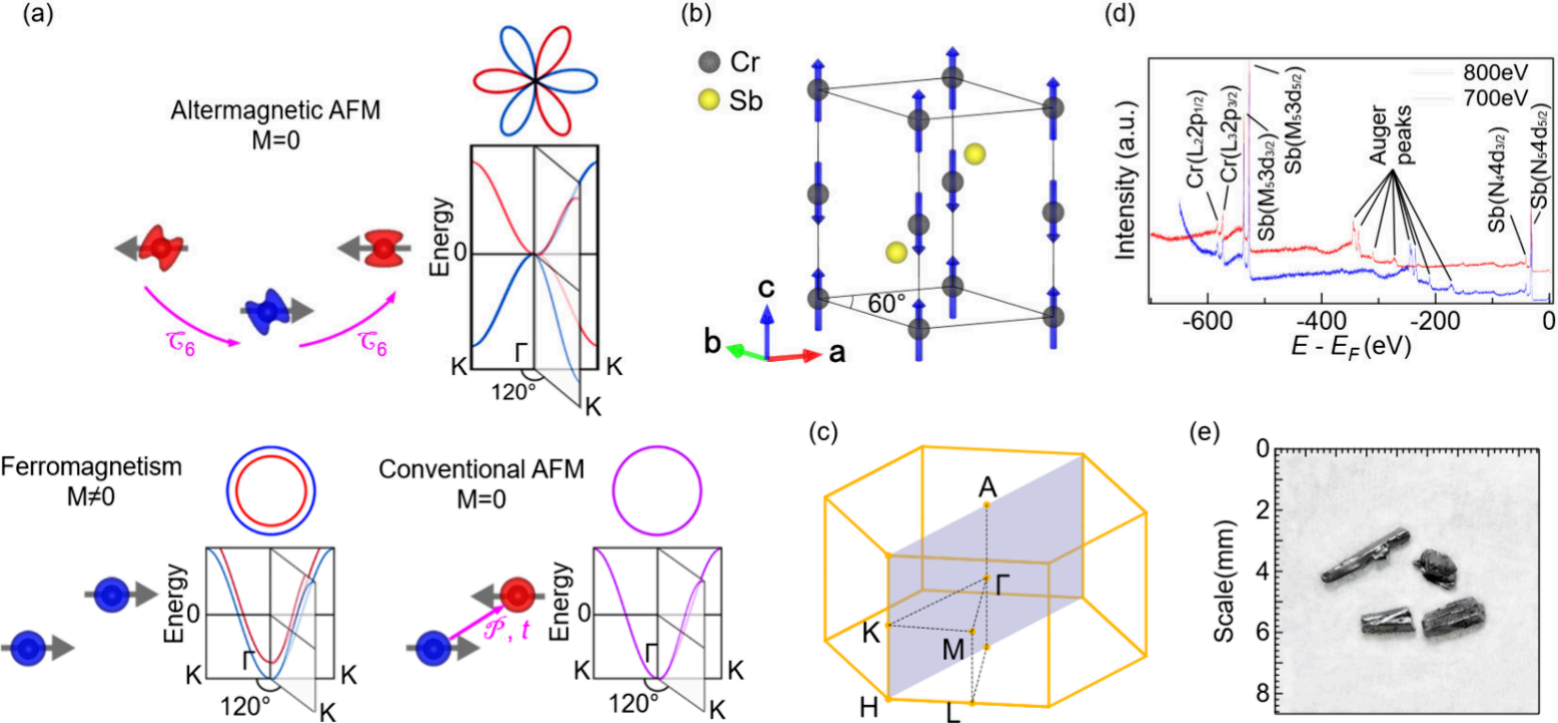
Basic information on CrSb. (a) The schematic illustrating the band spin degeneracy of altermagnetic, conventional AFM, and FM, respectively. The upper panel represents altermagnetism in hexagonal crystals, such as CrSb, in which the sublattices are correlated by the rotation (C_6*z*_) operation marked by pink arrows. (b) The crystal structure of the hexagonal CrSb. (c) The corresponding 3D BZ of CrSb, where the shadowed plane runs parallel to the cleavage (100) surface. (d) XPS of CrSb sample, in which each peak of corresponding elements is labeled. (e) The photography of the CrSb sample used for experiments.

The experimental results of overall bulk band dispersion acquired with soft X-ray along high symmetry lines agree well with the calculations under altermagnetic order in all directions instead of no-magnetic order, as shown in Figure 2a–d. For more details, please see the Supporting Information. As the cleavage is on the (100) surface, we assign the in-plane ΓK direction as *k*_*x*_, the in-plane ΓA direction as *k*_*y*_, and the out-of-plane ΓM direction as *k*_*z*_. Such a definition is different from the conventional ones but convenient for experimental notation. However, soft X-rays have the disadvantage of low energy resolution and statistics and are not sensitive to surface Fermi arcs in the presence of topologically nontrivial states. To address this, we conducted high energy resolution VUV ARPES experiments with photon energies ranging from 30 to 148 eV. The photon energy-dependent out-of-plane cut is plotted in Figure 2f, which agrees well with the band trend of calculation along the ΓM direction. We determined the high symmetry Γ planes corresponding to a photon energy of 102 eV and M planes at photon energies of 141 and 71 eV, which are consistent with the predictions based on the soft X-ray *k*_*z*_ data, as shown in Figure 2g.

**Figure 2:**
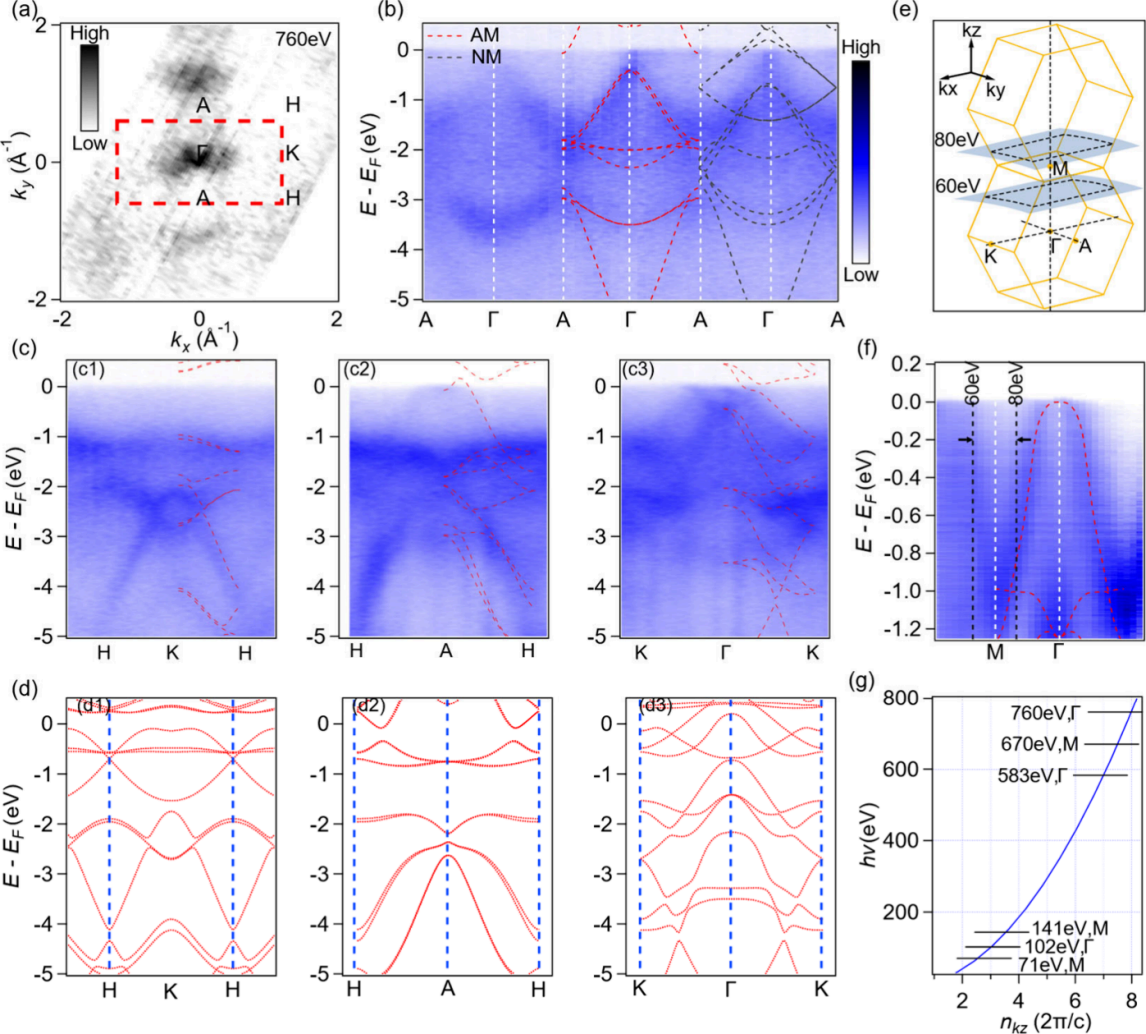
3D bulk electronic band structure of CrSb in general. (a) ARPES intensity of the Fermi surface at the K-Γ-A plane acquired with photon energy 760 eV. (b) Experimental band dispersion along the ΓA direction. The calculated band structures considering SOC under altermagnetic order (red dotted lines) and no-magnetic order (black dotted lines) are plotted alongside for comparison. (c) APRES intensity of cuts along KH, HA, and KΓ high symmetry lines, respectively. The calculated band structures considering SOC under altermagnetic order (red dotted lines) are plotted alongside for comparison. (d) Calculated band structure under the nonmagnetic phase considering SOC along the same high symmetry lines in (c). (e) 3D BZs of CrSb aligned vertically along *k*_*z*_ direction. (f) Photon energy dependent APRES intensity of the cut along the ΓM direction (*k*_*z*_). The black arrows indicate the substantial *k*_*z*_ broadening effect. The white dashed lines are the high symmetry momentum positions. (g) The photon energy dependent data fit yielded a relationship curve between photon energy and *k*_*z*_.

After discussing the 3D band structure, we now focus on band spin splitting, a crucial property for altermagnets. In CrSb, most of the bands along high-symmetry lines are spin double degenerate. To detect the spin splitting, measurements need to be better conducted along the generic momentum space. We selected two photon energies, 60 and 80 eV, as indicated by the gray planes in the 3D BZ in Figure 2e, where the corresponding planes in reciprocal space are both 0.3 times the distance of ΓM away from the M point (Figure 2e). The Fermi surfaces characterized by photon energies of 60 and 80 eV are shown in Figure 3a,b, respectively. For the 60 eV map, we took three cuts parallel to the ΓK direction from the second BZ, which are, respectively, evenly 0.075 times the distance of ΓA between each other away from the Γ point: cuts 1–3 (Figure 3d–f). For the 80 eV map, as the momentum space covers a larger area, we take the cuts with double separation of that from the 60 eV map as shown by cuts 4–5 in Figure 3g,h. We found from the experiments that the *k*_*z*_ broadening effect is very large at UV light range which is shown by the black arrows in Figure 2f. For each cut, the calculated band structure under magnetism, considering *k*_*z*_ broadening (plot with several sets of data under a range of *k*_*z*_), is shown (Figures 3i–m) for comparison with the ARPES spectra at 60 and 80 eV, respectively. In the ARPES intensity results for each cut, clear spin splitting up to 0.2 eV, noted by the corresponding red and blue arrows, is found to be consistent with the spin splitting in the calculated results. This indicates the presence of large spin splitting near the Fermi level under long-range altermagnetism in CrSb, which is also the evidence of parity-time symmetry breaking. In order to check whether the splitting is induced by the SOC effect, we plot the calculation considering SOC but without magnetic order in Figure S2 in the Supporting Information. It is shown that SOC does not induce any splitting in generic momentum positions. To isolate the potential influence of defects, we performed band structure calculations for systems containing Cr vacancies (Figure S3b) or excess Sb atoms (Figure S3c). When an enlarged unit cell is employed and the band structure is unfolded into the pristine BZ (Figure S3), our results reveal no evidence of spin splitting caused by such defects. Furthermore, energy-dispersive X-ray spectroscopy (EDS) analysis confirmed the stoichiometric ratio of Cr/Sb as 1:1 (Figure S4), aligning with the expected composition. Taken together, these findings strongly suggest that the observed spin splitting originates from altermagnetism rather than structural defects or compositional variations.

**Figure 3:**
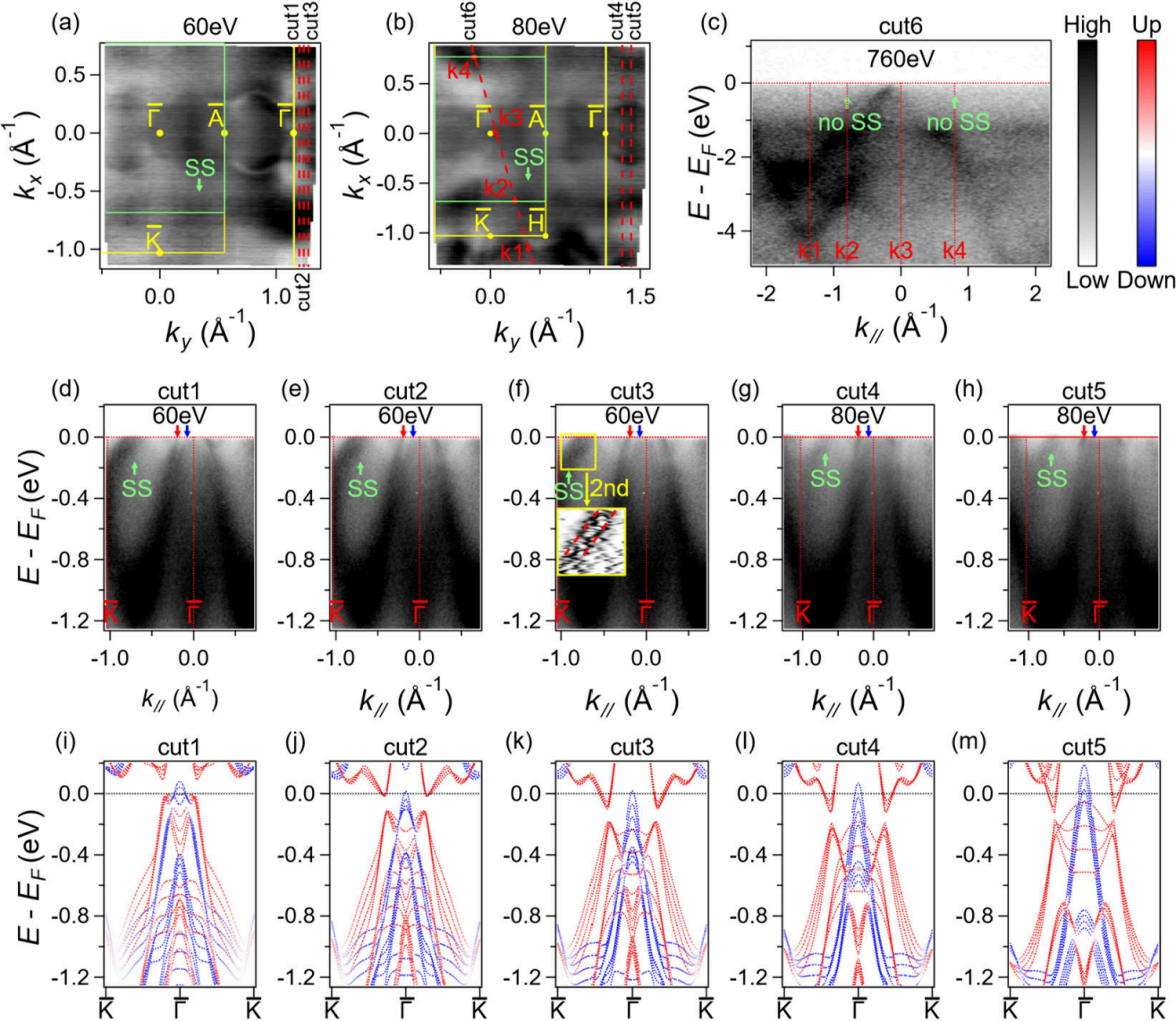
Experimental Fermi surfaces and band splitting of CrSb at arbitrary momentum space. (a, b) ARPES intensity of Fermi surfaces acquired with photon energies 60 and 80 eV, respectively. The yellow line and the green line represent Brillouin zone projection of bulk states and surface states, respectively. The red dotted lines indicate the positions of the cut 1–5 in the following panels. (c) Soft X-ray data along cut 6 without showing any surface bands at the position of the green arrow. (d–h) Experimental band dispersion along cuts 1–5. The red and blue arrows denote spin splitting. The green arrows denote the surface states. The subpanel in (f) is the curvature intensity plot of the area in the yellow box which reveals that the surface states consist of two bands. (i–m) Calculated band structure along cuts 1–5 with overlapping results from a range of *k*_*z*_ momentum to simulate *k*_*z*_ broadening in experiments.

Besides the bulk bands, we identified bands and Fermi surfaces that do not exist in the bulk band calculations, denoted by the green “SS” in Figure 3. These bands, located between the projections of Γ and K, form long Fermi surfaces, as shown in Figure 3a,b, centered at the surface BZ boundary as shallow hole-like bands indicated by green arrows in Figure 3d–h. When checking details with curvature intensity spectra, we identified two bands, as shown in the inset of Figure 3f. We attribute these bands to surface states, which is further confirmed by the photon energy dependent data, which show no *k*_*z*_ dispersion as shown in Figure S5, and by the absence of SS bands in the soft X-ray ARPES spectra shown in Figure 3c recorded with 760 eV photon energy.

From the band structure, we can easily identify band inversion and crossings near the Fermi level. Now, our analysis focuses on the topology of CrSb between bands *N* and *N* + 1 (where N is the occupation number) using the open-source software WannierTools.^45^ A preliminary search reveals numerous band crossings, which appear quite complex. In order to simplify these crossing nodes, we categorize them into two sets for spin-up and spin-down bands without SOC, and we observed seven nodal rings for each spin subset, indicated by red and blue colors in Figure 4a–c. Three nodal rings are located within the gray plane and its equivalents shown in Figure 4g between *k*_*y*_ = 0 and π/*c*, with 120° apart from each other and degenerate along the ΓZ high-symmetry line (please note that, in our notation, the *k*_*y*_ direction is parallel to the crystal’s *c* direction). Other three nodal rings are located between *k*_*y*_ = 0 and −π/*c*, which can be derived from the first set using the combination of M*_Y_* mirror and *C*_6*y*_ rotation operations. The seventh nodal ring is located near the *k*_*y*_ = 0 plane, exhibiting a wave-like out-of-plane shape. There exists a complex conjugation symmetry, denoted as *C* (where *C* = *K*), analogous to the time-reversal symmetry in spinless systems. Consequently, the combined antiunitary symmetry of (*C*) and the inversion symmetry (*I*) satisfies (*CI*)^2^ = 1, which imposes a constraint that forbids one of the three Pauli matrices in a two-band effective model at any given k-point. This condition ensures the presence of the seventh nodal ring, as described by the topological invariant .^46^ The nodal rings for spin-up and spin-down crossing nodes can be related by the M*_Y_* mirror operation. When SOC is considered, the nodal rings open gaps except for 12 Weyl points with green spheres representing nodes with chirality ξ = +1 and pink spheres representing ξ = −1, symmetrically located at *k*_*y*_ = ±0.796π/c planes, as shown in Figure 4d–f. The positions of Weyl points with ξ = +1 are listed in Table 1, and the positions of Weyl points with ξ = −1 can be calculated with opposite *k*_*x*_ and *k*_*z*_. We plot the band structure along a cut that intersects two Weyl points, marked as −*M*_*W*_-Γ_*W*_-*M*_*W*_ (*k*_*y*_ = 0.796π/*c*) in Figure 4g. The calculated band structures, both without and with SOC, are displayed in Figure 4h, showing the band crossings for the nodal rings and Weyl points.

**Figure 4:**
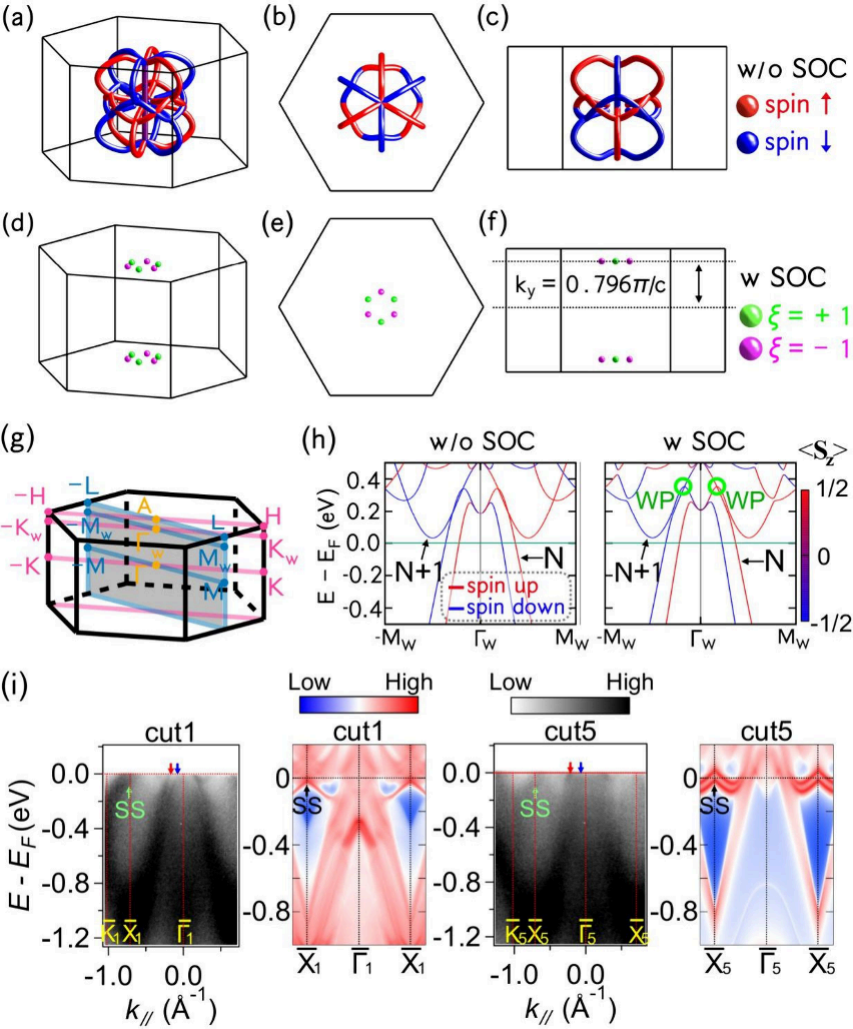
(a–c) The distribution of the nodal line for bands with occupation numbers *N* and *N* + 1 (as indicated in (h)) in the first Brillouin zone without SOC, with red (blue) color representing points with spin up (down). (d–f) The distribution of the Weyl points with SOC, with green (pink) spheres representing nodes with chirality ξ = +1 (−1). (g) The 3D BZ of the primitive cell of CrSb. (h) Spin splitting bulk bands along the path −*M*_*W*_-Γ*_W_*-*M*_*W*_ (*k*_*y*_ = 0.796π/*c*) are shown both without and with SOC. The regions highlighted by green circles, indicating the positions of the Weyl points. (i) Comparison of surface states between experimental results and calculated surface projected band structure with Cr termination along different cuts.

**Table 1:** Positions of Weyl Points in the 3D BZ

		*k*_*y*_/(π/c)	
WP1	0	±0.796	0.188
WP2	0.094	±0.796	–0.162
WP3	–0.094	±0.796	–0.162

After discussion the topology of bulk bands, we now focus on the surface states. The (100) surface is a natural cleavage plane. We calculated the band structure along this surface and compared it with the experimental results along different cuts, as shown in Figure 4i. The observed surface states agree well with that of the calculation. In order to check the topology of the surface states, we extend the calculated structure to 20-unit cell and plot the surface projected band structure and Fermi surfaces in Figure S3. As the surface potential in reality can be different, the dispersion of surface bands between experiments and calculations always has some differences in detail. Since the projections of Weyl nodes merge in the bulk band projection, it is challenging to determine how the surface Fermi arcs connect with the Weyl nodes. However, from the trend of the surface bands in the middle of Figure S6a, it appears that the two brunch surface Fermi arcs terminate toward the Weyl nodes. On the (100) cleavage surface, two Weyl nodes overlap, resulting in two branches of surface Fermi arcs, consistent with the double surface band structure. The double surface states can be viewed in cut 5 in the calculation as shown in Figure 4i, which agree with our experimental observation as shown in Figure 3. The surface state calculations presented in Figure 4 and Figure S6 are performed for the Cr-terminated surface, demonstrating strong agreement between experimental and theoretical results. In contrast, additional calculations for Sb-terminated surfaces (Figure S7) reveal marked discrepancies compared to the experimental data, strongly suggesting that the cleaved surface adopts Cr termination. Furthermore, trivial surface states originating from the nonmagnetic phase of CrSb were analyzed (Figure S8), which exhibit stark contrast with our experimental observations. These findings collectively confirm that the observed surface states arise from the altermagnetic Weyl phase rather than nonmagnetic contributions.

In summary, we investigated the complex band structure of altermagnet CrSb by utilizing both UV ARPES and soft X-ray ARPES experiments. We observed a prominent spin-split 3D bulk band structure, proving the predictions of LDA calculations for g-wave altermagnetism. Furthermore, we observed surface bands on the (100) cleavage that have not been reported in previous studies. Our further analysis by calculations indicates that these surface states may originate from the bulk topological Weyl nodes, which contribute to the unique nontrivial band topology of CrSb. The breaking of parity-time symmetry is crucial in fostering the novel states of altermagnetism and topology within CrSb. This synergy has the potential to enhance the future possible functionality of spintronic devices and expand their diverse applications by combining two sets of unusual physical features such as topology and altermagnetism in a single material.

## Methods

The crystals of CrSb were grown by a self-flux method. Cr and Sb with atomic ratios of 1:4 were loaded in an alumina crucible and sealed in an evacuated quartz tube. The tube was heated to 1000 °C, kept for 20 h, and then cooled to 750 °C with a rate of 2 °C/h. After that, the sample was taken out of the furnace and centrifuged to separate the crystals from flux. Conventional ARPES measurements were performed at the beamline UE112 PGM-2b-1^2 of BESSY (Berlin Electron Storage Ring Society for Synchrotron Radiation) synchrotron, at Bloch beamline of MAX-IV synchrotron, and at BL03U beamline of the Shanghai Synchrotron Radiation Facility (SSRF). The energy and angular resolutions were set to ∼20 meV and 0.1°, respectively, and the temperature was set to around 20 K. Soft X-ray ARPES measurements were performed at BL25SU beamline of SPring-8 Synchrotron, and the temperature was set to around 77 K. The samples for all ARPES measurements were cleaved in situ and measured in a vacuum better than 2 × 10^–10^ Torr. We have calculated the ground state electronic structure of CrSb. Bulk band calculations were performed within the Perdew-Burke-Ernzernhof (PBE)^47^ generalized gradient approximation using the QUANTUM ESPRESSO package^48,49^ with ultrasoft pseudopotential from the PSLibrary.^50^ The kinetic energy cutoff for wave functions is 80 Ry with a charge density cutoff of 640 Ry. A Monkhorst–Pack 12 × 12 × 10 *k*-mesh has been used.^51^ For topological band structure calculations in Figure 4, we conducted the ab initio electronic structure calculations for CrSb using the pseudopotential Vienna Ab initio Simulation Package (VASP)^52^ with the Perdew–Burke–Ernzerhof (PBE) generalized gradient approximation (GGA).^47^ For the bulk band calculation, a 11 × 11 × 9 k-grid, an energy cutoff of 400 eV, and an energy convergence criterion of 10^–7^ eV were employed. For the slab band calculation, a 9 × 6 × 1 k-grid, an energy cutoff of 400 eV, and an energy convergence criterion of 10^–5^ eV were employed. To study the topology of CrSb between bands *N* and *N* + 1, as well as edge states and the Fermi surface along (100), we determined maximally localized Wannier functions using a reduced basis set formed by the d orbitals of Cr and p orbitals of Sb atoms in the Wannier90 software^53^ and then used wannhr_symm_Mag^54^ to symmetrize the real-space Hamiltonian. The theoretical simulations were conducted using the WannierTools package.^45^ The lattice parameters of the primitive cell are as follows: *a* = *b* = 4.103 Å and *c* = 5.463 Å.

## Data Availability

The authors declare that all the relevant data are available within the paper and its Supporting Information file or from the corresponding author upon reasonable request.

## Supporting Information Available

Additional data and discussion about the crystal quality, physical properties, experimental and calculated band structure of altermagnetism in CrSb: Section 1: Discussion about the properties of altermagnetism; Section 2: General agreement between experimental bulk band structure and calculations; Supplementary Figures 1–8 including: the XRD data, EDS data to show the sample quality; SOC effect on the band structure; the effect of defects on band structure; the *k*_*z*_ dependence of band structure and surface termination analysis (PDF)

## References

[ref1] Wolf S. A.; Awschalom D. D.; Buhrman R. A.; Daughton J. M.; von Molnár S.; Roukes M. L.; Chtchelkanova A. Y.; Treger D. M. Spintronics: A Spin-Based Electronics Vision for the Future. Science (1979) 2001, 294 (5546), 1488–1495. 10.1126/science.1065389. 11711666

[ref2] Žutić I.; Fabian J.; Das Sarma S. Spintronics: Fundamentals and Applications. Rev. Mod. Phys. 2004, 76 (2), 323–410. 10.1103/RevModPhys.76.323.

[ref3] THOMAS L. H. The Motion of the Spinning Electron. Nature 1926, 117 (2945), 514–514. 10.1038/117514a0.

[ref4] Yuan L.-D.; Wang Z.; Luo J.-W.; Rashba E. I.; Zunger A. Giant Momentum-Dependent Spin Splitting in Centrosymmetric Low-*Z* Antiferromagnets. Phys. Rev. B 2020, 102 (1), 014422 10.1103/PhysRevB.102.014422.

[ref5] Hayami S.; Yanagi Y.; Kusunose H. Momentum-Dependent Spin Splitting by Collinear Antiferromagnetic Ordering. J. Phys. Soc. Jpn. 2019, 88 (12), 123702 10.7566/JPSJ.88.123702.

[ref6] Hayami S.; Yanagi Y.; Kusunose H. Bottom-up Design of Spin-Split and Reshaped Electronic Band Structures in Antiferromagnets without Spin-Orbit Coupling: Procedure on the Basis of Augmented Multipoles. Phys. Rev. B 2020, 102 (14), 144441 10.1103/PhysRevB.102.144441.

[ref7] Šmejkal L.; González-Hernández R.; Jungwirth T.; Sinova J. Crystal Time-Reversal Symmetry Breaking and Spontaneous Hall Effect in Collinear Antiferromagnets. Sci. Adv. 2020, 6 (23), eaaz8809 10.1126/sciadv.aaz8809. 32548264 PMC7274798

[ref8] Mazin I. I.; Koepernik K.; Johannes M. D.; González-Hernández R.; Šmejkal L. Prediction of Unconventional Magnetism in Doped FeSb2. Proc. Natl. Acad. Sci. U. S. A. 2021, 118 (42), e2108924118 10.1073/pnas.2108924118. 34649995 PMC8594493

[ref9] González-Hernández R.; Šmejkal L.; Výborný K.; Yahagi Y.; Sinova J.; Jungwirth T.; Železný J. Efficient Electrical Spin Splitter Based on Nonrelativistic Collinear Antiferromagnetism. Phys. Rev. Lett. 2021, 126 (12), 127701 10.1103/PhysRevLett.126.127701. 33834809

[ref10] Shao D.-F.; Zhang S.-H.; Li M.; Eom C.-B.; Tsymbal E. Y. Spin-Neutral Currents for Spintronics. Nat. Commun. 2021, 12 (1), 7061 10.1038/s41467-021-26915-3. 34862380 PMC8642435

[ref11] Ma H.-Y.; Hu M.; Li N.; Liu J.; Yao W.; Jia J.-F.; Liu J. Multifunctional Antiferromagnetic Materials with Giant Piezomagnetism and Noncollinear Spin Current. Nat. Commun. 2021, 12 (1), 2846 10.1038/s41467-021-23127-7. 33990597 PMC8121910

[ref12] Šmejkal L.; MacDonald A. H.; Sinova J.; Nakatsuji S.; Jungwirth T. Anomalous Hall Antiferromagnets. Nat. Rev. Mater. 2022, 7 (6), 482–496. 10.1038/s41578-022-00430-3.

[ref13] Liu P.; Li J.; Han J.; Wan X.; Liu Q. Spin-Group Symmetry in Magnetic Materials with Negligible Spin-Orbit Coupling. Phys. Rev. X 2022, 12 (2), 021016 10.1103/PhysRevX.12.021016.

[ref14] Naka M.; Hayami S.; Kusunose H.; Yanagi Y.; Motome Y.; Seo H. Spin Current Generation in Organic Antiferromagnets. Nat. Commun. 2019, 10 (1), 4305 10.1038/s41467-019-12229-y. 31541112 PMC6754401

[ref15] Šmejkal L.; Sinova J.; Jungwirth T. Emerging Research Landscape of Altermagnetism. Phys. Rev. X 2022, 12 (4), 040501 10.1103/PhysRevX.12.040501.

[ref16] Šmejkal L.; Sinova J.; Jungwirth T. Beyond Conventional Ferromagnetism and Antiferromagnetism: A Phase with Nonrelativistic Spin and Crystal Rotation Symmetry. Phys. Rev. X 2022, 12 (3), 031042 10.1103/PhysRevX.12.031042.

[ref17] Feng Z.; Zhou X.; Šmejkal L.; Wu L.; Zhu Z.; Guo H.; González-Hernández R.; Wang X.; Yan H.; Qin P.; Zhang X.; Wu H.; Chen H.; Meng Z.; Liu L.; Xia Z.; Sinova J.; Jungwirth T.; Liu Z. An Anomalous Hall Effect in Altermagnetic Ruthenium Dioxide. Nat. Electron 2022, 5 (11), 735–743. 10.1038/s41928-022-00866-z.

[ref18] Bai H.; Zhang Y. C.; Zhou Y. J.; Chen P.; Wan C. H.; Han L.; Zhu W. X.; Liang S. X.; Su Y. C.; Han X. F.; Pan F.; Song C. Efficient Spin-to-Charge Conversion via Altermagnetic Spin Splitting Effect in Antiferromagnet RuO2. Phys. Rev. Lett. 2023, 130 (21), 216701 10.1103/PhysRevLett.130.216701. 37295074

[ref19] Mazin I. I. Altermagnetism in MnTe: Origin, Predicted Manifestations, and Routes to Detwinning. Phys. Rev. B 2023, 107 (10), L100418 10.1103/PhysRevB.107.L100418.

[ref20] Fedchenko O.; Minár J.; Akashdeep A.; D’Souza S. W.; Vasilyev D.; Tkach O.; Odenbreit L.; Nguyen Q.; Kutnyakhov D.; Wind N.; Wenthaus L.; Scholz M.; Rossnagel K.; Hoesch M.; Aeschlimann M.; Stadtmüller B.; Kläui M.; Schönhense G.; Jungwirth T.; Hellenes A. B.; Jakob G.; Šmejkal L.; Sinova J.; Elmers H.-J. Observation of Time-Reversal Symmetry Breaking in the Band Structure of Altermagnetic RuO2. Sci. Adv. 2024, 10 (5), eadj4883 10.1126/sciadv.adj4883. 38295181 PMC10830110

[ref21] Reimers S.; Odenbreit L.; Šmejkal L.; Strocov V. N.; Constantinou P.; Hellenes A. B.; Jaeschke Ubiergo R.; Campos W. H.; Bharadwaj V. K.; Chakraborty A.; Denneulin T.; Shi W.; Dunin-Borkowski R. E.; Das S.; Kläui M.; Sinova J.; Jourdan M. Direct Observation of Altermagnetic Band Splitting in CrSb Thin Films. Nat. Commun. 2024, 15 (1), 2116 10.1038/s41467-024-46476-5. 38459058 PMC10923844

[ref22] Zhou Z.; Cheng X.; Hu M.; Liu J.; Pan F.; Song C. Crystal Design of Altermagnetism. arXiv 2024, 2403.07396 10.48550/arXiv.2403.07396.

[ref23] Lin Z.; Chen D.; Lu W.; Liang X.; Feng S.; Yamagami K.; Osiecki J.; Leandersson M.; Thiagarajan B.; Liu J.; Felser C.; Ma J. Observation of Giant Spin Splitting and D-Wave Spin Texture in Room Temperature Altermagnet RuO2. arXiv 2024, 2402.04995 10.48550/arXiv.2402.04995.

[ref24] Bai L.; Feng W.; Liu S.; Šmejkal L.; Mokrousov Y.; Yao Y. Altermagnetism: Exploring New Frontiers in Magnetism and Spintronics. Adv. Funct Mater. 2024, 34, 2409327 10.1002/adfm.202409327.

[ref25] Hu M.; Cheng X.; Huang Z.; Liu J. Catalogue of C-paired Spin-Momentum Locking in Antiferromagnetic Systems. arXiv 2024, 2407.02319 10.48550/arXiv.2407.02319.

[ref26] Armitage N. P.; Mele E. J.; Vishwanath A. Weyl and Dirac Semimetals in Three-Dimensional Solids. Rev. Mod. Phys. 2018, 90 (1), 015001 10.1103/RevModPhys.90.015001.

[ref27] Wan X.; Turner A. M.; Vishwanath A.; Savrasov S. Y. Topological Semimetal and Fermi-Arc Surface States in the Electronic Structure of Pyrochlore Iridates. Phys. Rev. B 2011, 83 (20), 205101 10.1103/PhysRevB.83.205101.

[ref28] Wang Z.; Sun Y.; Chen X.-Q.; Franchini C.; Xu G.; Weng H.; Dai X.; Fang Z. Dirac Semimetal and Topological Phase Transitions in *A*3 Bi (*A*=Na, K, Rb). Phys. Rev. B 2012, 85 (19), 195320 10.1103/PhysRevB.85.195320.

[ref29] Weng H.; Fang C.; Fang Z.; Bernevig B. A.; Dai X. Weyl Semimetal Phase in Noncentrosymmetric Transition-Metal Monophosphides. Phys. Rev. X 2015, 5 (1), 011029 10.1103/PhysRevX.5.011029.

[ref30] Huang S.-M.; Xu S.-Y.; Belopolski I.; Lee C.-C.; Chang G.; Wang B.; Alidoust N.; Bian G.; Neupane M.; Zhang C.; Jia S.; Bansil A.; Lin H.; Hasan M. Z. A Weyl Fermion Semimetal with Surface Fermi Arcs in the Transition Metal Monopnictide TaAs Class. Nat. Commun. 2015, 6 (1), 7373 10.1038/ncomms8373. 26067579 PMC4490374

[ref31] Liu Z. K.; Zhou B.; Zhang Y.; Wang Z. J.; Weng H. M.; Prabhakaran D.; Mo S.-K.; Shen Z. X.; Fang Z.; Dai X.; Hussain Z.; Chen Y. L. Discovery of a Three-Dimensional Topological Dirac Semimetal, Na3 Bi. Science (1979) 2014, 343 (6173), 864–867. 10.1126/science.1245085. 24436183

[ref32] Lv B. Q.; Weng H. M.; Fu B. B.; Wang X. P.; Miao H.; Ma J.; Richard P.; Huang X. C.; Zhao L. X.; Chen G. F.; Fang Z.; Dai X.; Qian T.; Ding H. Experimental Discovery of Weyl Semimetal TaAs. Phys. Rev. X 2015, 5 (3), 031013 10.1103/PhysRevX.5.031013.

[ref33] Xu S.-Y.; Belopolski I.; Alidoust N.; Neupane M.; Bian G.; Zhang C.; Sankar R.; Chang G.; Yuan Z.; Lee C.-C.; Huang S.-M.; Zheng H.; Ma J.; Sanchez D. S.; Wang B.; Bansil A.; Chou F.; Shibayev P. P.; Lin H.; Jia S.; Hasan M. Z. Discovery of a Weyl Fermion Semimetal and Topological Fermi Arcs. Science (1979) 2015, 349 (6248), 613–617. 10.1126/science.aaa9297. 26184916

[ref34] Liu D. F.; Liang A. J.; Liu E. K.; Xu Q. N.; Li Y. W.; Chen C.; Pei D.; Shi W. J.; Mo S. K.; Dudin P.; Kim T.; Cacho C.; Li G.; Sun Y.; Yang L. X.; Liu Z. K.; Parkin S. S. P.; Felser C.; Chen Y. L. Magnetic Weyl Semimetal Phase in a Kagomé Crystal. Science (1979) 2019, 365 (6459), 1282–1285. 10.1126/science.aav2873. 31604236

[ref35] Zhan J.; Li J.; Shi W.; Chen X.-Q.; Sun Y. Coexistence of Weyl Semimetal and Weyl Nodal Loop Semimetal Phases in a Collinear Antiferromagnet. Phys. Rev. B 2023, 107 (22), 224402 10.1103/PhysRevB.107.224402.

[ref36] Zhou X.; Feng W.; Zhang R.-W.; Šmejkal L.; Sinova J.; Mokrousov Y.; Yao Y. Crystal Thermal Transport in Altermagnetic RuO2. Phys. Rev. Lett. 2024, 132 (5), 056701 10.1103/PhysRevLett.132.056701. 38364129

[ref37] Antonenko D. S.; Fernandes R. M.; Venderbos J. W. F. Mirror Chern Bands and Weyl Nodal Loops in Altermagnets. Phys. Rev. Lett. 2025, 134 (7), 096703 10.1103/PhysRevLett.134.096703. 40131055

[ref38] Krempaský J.; Šmejkal L.; D’Souza S. W.; Hajlaoui M.; Springholz G.; Uhlířová K.; Alarab F.; Constantinou P. C.; Strocov V.; Usanov D.; Pudelko W. R.; González-Hernández R.; Birk Hellenes A.; Jansa Z.; Reichlová H.; Šobáň Z.; Gonzalez Betancourt R. D.; Wadley P.; Sinova J.; Kriegner D.; Minár J.; Dil J. H.; Jungwirth T. Altermagnetic Lifting of Kramers Spin Degeneracy. Nature 2024, 626 (7999), 517–522. 10.1038/s41586-023-06907-7. 38356066 PMC10866710

[ref39] Zhu Y.-P.; Chen X.; Liu X.-R.; Liu Y.; Liu P.; Zha H.; Qu G.; Hong C.; Li J.; Jiang Z.; Ma X.-M.; Hao Y.-J.; Zhu M.-Y.; Liu W.; Zeng M.; Jayaram S.; Lenger M.; Ding J.; Mo S.; Tanaka K.; Arita M.; Liu Z.; Ye M.; Shen D.; Wrachtrup J.; Huang Y.; He R.-H.; Qiao S.; Liu Q.; Liu C. Observation of Plaid-like Spin Splitting in a Noncoplanar Antiferromagnet. Nature 2024, 626 (7999), 523–528. 10.1038/s41586-024-07023-w. 38356068

[ref40] Yang G.; Li Z.; Yang S.; Li J.; Zheng H.; Zhu W.; Cao S.; Zhao W.; Zhang J.; Ye M.; Song Y.; Hu L.-H.; Yang L.; Shi M.; Yuan H.; Zhang Y.; Xu Y.; Liu Y. Three-Dimensional Mapping of the Altermagnetic Splitting in CrSb. Nat. Commun. 2025, 16 (1), 1442 10.1038/s41467-025-56647-7. 39920139 PMC11805911

[ref41] Ding J.; Jiang Z.; Chen X.; Tao Z.; Liu Z.; Liu J.; Li T.; Liu J.; Yang Y.; Zhang R.; Deng L.; Jing W.; Huang Y.; Shi Y.; Qiao S.; Wang Y.; Guo Y.; Feng D.; Shen D. Large Band Splitting in g-Wave Altermagnet CrSb. Phys. Rev. Lett. 2024, 133 (7), 206401 10.1103/PhysRevLett.133.206401. 39626706

[ref42] Zeng M.; Zhu M.; Zhu Y.; Liu X.; Ma X.; Hao Y.; Liu P.; Qu G.; Yang Y.; Jiang Z.; Yamagami K.; Arita M.; Zhang X.; Shao T.; Dai Y.; Shimada K.; Liu Z.; Ye M.; Huang Y.; Liu Q.; Liu C. Observation of Spin Splitting in Room-Temperature Metallic Antiferromagnet CrSb. Adv. Sci. 2024, 11, 2406529 10.1002/advs.202406529. PMC11578308 39303163

[ref43] Li C.; Hu M.; Li Z.; Wang Y.; Chen W.; Thiagarajan B.; Leandersson M.; Polley C.; Kim T.; Liu H.; Fulga C.; Vergniory M. G.; Janson O.; Tjernberg O.; Brink J. Topological Weyl Altermagnetism in CrSb. arXiv 2024, 2405.14777 10.48550/arXiv.2405.14777.

[ref44] Takei W. J.; Cox D. E.; Shirane G. Magnetic Structures in the MnSb-CrSb System. Phys. Rev. 1963, 129 (5), 2008–2018. 10.1103/PhysRev.129.2008.

[ref45] Wu Q.; Zhang S.; Song H.-F.; Troyer M.; Soluyanov A. A. WannierTools: An Open-Source Software Package for Novel Topological Materials. Comput. Phys. Commun. 2018, 224, 405–416. 10.1016/j.cpc.2017.09.033.

[ref46] Xu Y.; Song Z.; Wang Z.; Weng H.; Dai X. Higher-Order Topology of the Axion Insulator EuIn2As2. Phys. Rev. Lett. 2019, 122 (25), 256402 10.1103/PhysRevLett.122.256402. 31347874

[ref47] Perdew J. P.; Burke K.; Ernzerhof M. Generalized Gradient Approximation Made Simple. Phys. Rev. Lett. 1996, 77 (18), 3865–3868. 10.1103/PhysRevLett.77.3865. 10062328

[ref48] Giannozzi P.; Andreussi O.; Brumme T.; Bunau O.; Buongiorno Nardelli M.; Calandra M.; Car R.; Cavazzoni C.; Ceresoli D.; Cococcioni M.; Colonna N.; Carnimeo I.; Dal Corso A.; de Gironcoli S.; Delugas P.; DiStasio R. A.; Ferretti A.; Floris A.; Fratesi G.; Fugallo G.; Gebauer R.; Gerstmann U.; Giustino F.; Gorni T.; Jia J.; Kawamura M.; Ko H.-Y.; Kokalj A.; Küçükbenli E.; Lazzeri M.; Marsili M.; Marzari N.; Mauri F.; Nguyen N. L.; Nguyen H.-V.; Otero-de-la-Roza A.; Paulatto L.; Poncé S.; Rocca D.; Sabatini R.; Santra B.; Schlipf M.; Seitsonen A. P.; Smogunov A.; Timrov I.; Thonhauser T.; Umari P.; Vast N.; Wu X.; Baroni S. Advanced Capabilities for Materials Modelling with Quantum ESPRESSO. J. Phys.: Condens. Matter 2017, 29 (46), 465901 10.1088/1361-648X/aa8f79. 29064822

[ref49] Giannozzi P.; Baroni S.; Bonini N.; Calandra M.; Car R.; Cavazzoni C.; Ceresoli D.; Chiarotti G. L.; Cococcioni M.; Dabo I.; Dal Corso A.; de Gironcoli S.; Fabris S.; Fratesi G.; Gebauer R.; Gerstmann U.; Gougoussis C.; Kokalj A.; Lazzeri M.; Martin-Samos L.; Marzari N.; Mauri F.; Mazzarello R.; Paolini S.; Pasquarello A.; Paulatto L.; Sbraccia C.; Scandolo S.; Sclauzero G.; Seitsonen A. P.; Smogunov A.; Umari P.; Wentzcovitch R. M. QUANTUM ESPRESSO: A Modular and Open-Source Software Project for Quantum Simulations of Materials. J. Phys.: Condens. Matter 2009, 21 (39), 395502 10.1088/0953-8984/21/39/395502. 21832390

[ref50] Dal Corso A. Pseudopotentials Periodic Table: From H to Pu. Comput. Mater. Sci. 2014, 95, 337–350. 10.1016/j.commatsci.2014.07.043.

[ref51] Monkhorst H. J.; Pack J. D. Special Points for Brillouin-Zone Integrations. Phys. Rev. B 1976, 13 (12), 5188–5192. 10.1103/PhysRevB.13.5188.

[ref52] Kresse G.; Furthmüller J. Efficient Iterative Schemes for Ab Initio Total-Energy Calculations Using a Plane-Wave Basis Set. Phys. Rev. B 1996, 54 (16), 11169–11186. 10.1103/PhysRevB.54.11169. 9984901

[ref53] Mostofi A. A.; Yates J. R.; Lee Y.-S.; Souza I.; Vanderbilt D.; Marzari N. Wannier90: A Tool for Obtaining Maximally-Localised Wannier Functions. Comput. Phys. Commun. 2008, 178 (9), 685–699. 10.1016/j.cpc.2007.11.016.

[ref54] Yue C. Wannhr_symm_Mag: A Tool for Symmetrization of Magnetic WannierTB; https://github.com/quanshengwu/wannier_tools/tree/master/utility/wannhr_symm_Mag (accessed 2025–4–22).

