# Supplementary material for: Signature of Topological Surface Bands in Altermagnetic Weyl Semimetal CrSb
Source: Nano Lett. 2025 Apr 28;25(18):7343–50. doi: 10.1021/acs.nanolett.5c00482 (PMC12063174; doi:10.1021/acs.nanolett.5c00482)
Supplement: Supplementary file 1 — nl5c00482_si_001.pdf [file nl5c00482_si_001.pdf]

Supporting Information for

Signature of Topological Surface Bands in Altermagnetic Weyl Semimetal CrSb

Wenlong Lu, Shiyu Feng, Yuzhi Wang, Dong Chen, Zihan Lin, Xin Liang, Siyuan Liu, Wanxiang Feng, Kohei Yamagami, Junwei Liu, Claudia Felser, Quansheng Wu, Junzhang Ma

This file contains:

1, Section 1: Discussion about the properties of altermagnetism

2, Section 2: General agreement between experimental bulk band structure and calculations

3, Supplementary Figure 1-8

Section 1: Discussion about the properties of altermagnetism

Altermagnetism enables unconventional high even-parity wave magnetism, such as d-wave, g-wave, and i-wave, to exist within the framework of an effective single-particle description of magnetism[1–3]. Recently, it has even been suggested that altermagnetism can extend to include non-collinear spins and multiple local-structure variations with odd-parity spin symmetry[4]. The study of altermagnetism significantly broadens the symmetry classification in the field of magnetism. Altermagnets are promising candidates for driving the next generation of information technology because of their unique physical properties: C-paired spin-valley locking, nontrivial Berry phase, spin currents, the anomalous Hall effect, stability under perturbing magnetic fields, and giant magnetoresistance, etc[1,5–18].

Section 2: General agreement between experimental bulk band structure and calculations

To confirm the agreement between the bulk experimental band structure and the theoretical calculations, we conducted a systematic soft X-ray ARPES study on CrSb. We cleaved the sample along the (100) side surface. Photon energies versus k_z were easily determined using soft X-ray ARPES data as the k_z resolution is much higher compared with that of low photon energies, which clearly reveals the periodic structure. This periodicity aligns well with the lattice parameter along the normal direction, allowing us to identify the Γ plane at 760 eV and 583 eV, the M plane at 670 eV. The in-plane Fermi surface along the ΓAHK plane is shown in Figure 2(a), with a rectangular BZ acquired using a photon energy of 760 eV. The photon energy corresponding to the Γ point was confirmed by scanning photon energy and characterizing along the ΓM direction (k_z) in momentum space. We present cuts along the high-symmetry lines ΓA, HK, HA, and ΓK in Figure 2(b) and 2(c), respectively. The calculated band structure under altermagnetic order is plotted alongside for comparison, indicated by red dotted lines. It can be seen that the experimental results agree well with the calculations in all directions. We verified that the materials are under altermagnetic order by plotting the band structure calculations for the non-magnetic

order as a reference, indicated by red dotted lines in Figure. 2(b) and (d), since the magnetic phase transition temperature exceeds ~700 K, which is beyond our experimental setting. The material retains long-range magnetic order under the conditions of our experiment, as indicated by the non-magnetic order calculations not matching the experimental data.

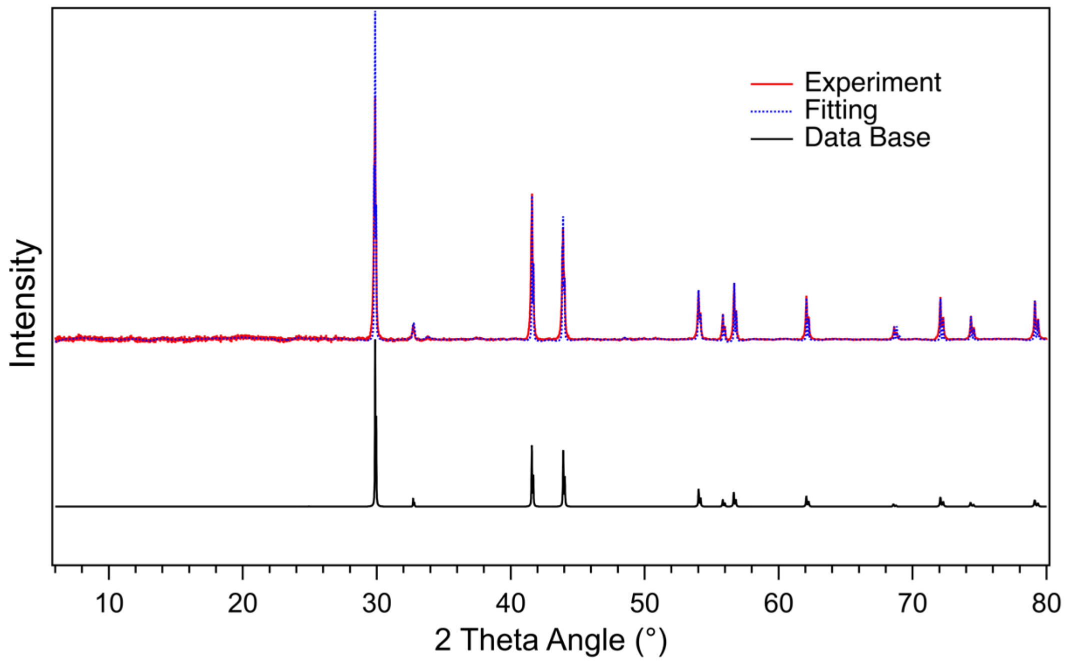

Figure S1. X-ray diffraction of CrSb crystal and the fitting results, compared with default database measured with Cu K alpha x-ray.

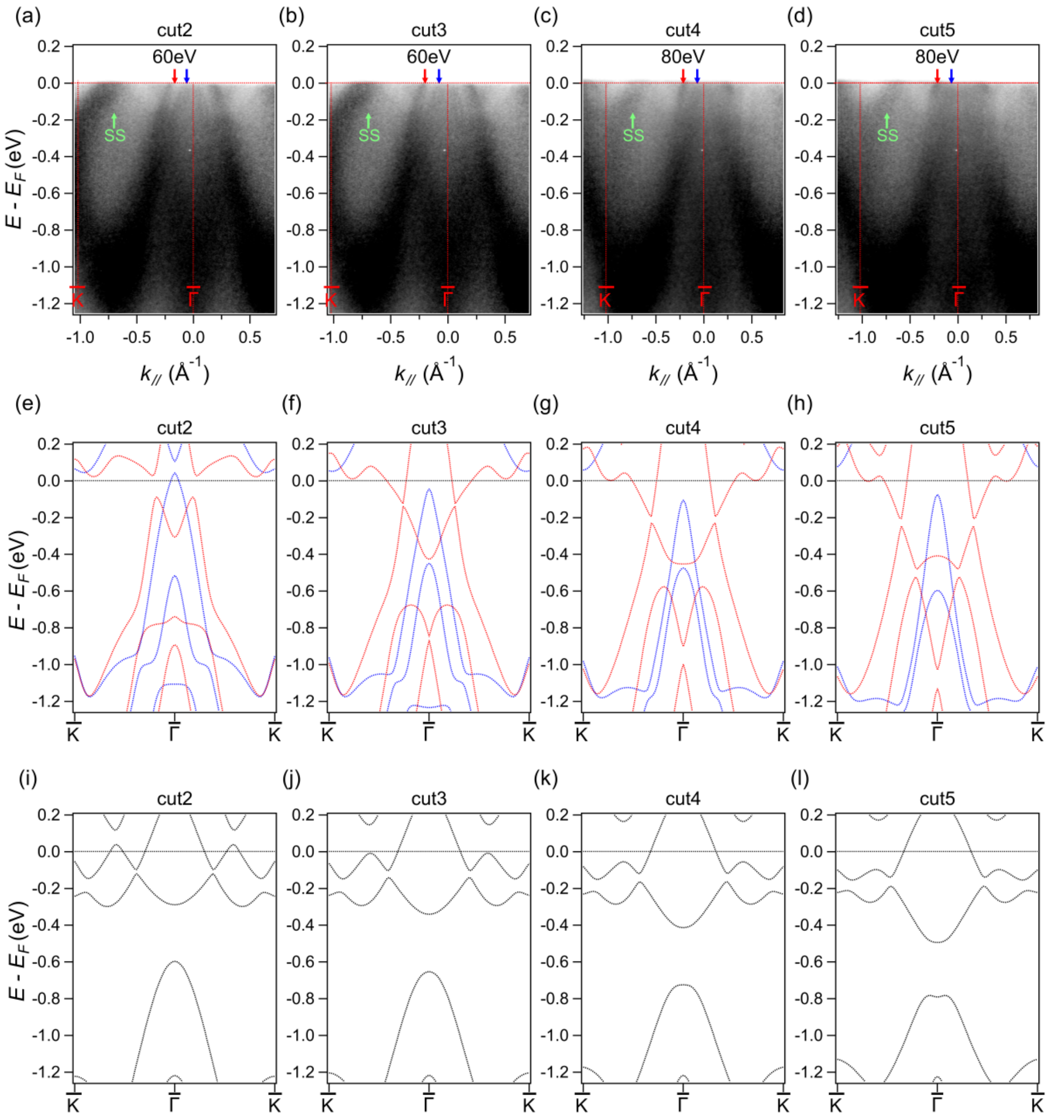

Figure S2. Comparison along different cuts among experimental results, calculated results concerning AFM without SOC and calculated results concerning SOC without magnetism, indicating the splitting is caused by AFM instead of SOC. (a-d) Experimental band dispersion along cut2-5, which are the same as that shown in Figure 3. (e-h) Calculated results along cut2-5 concerning AFM without SOC. (i-l) Calculated results along cut2-5 concerning SOC without magnetism.

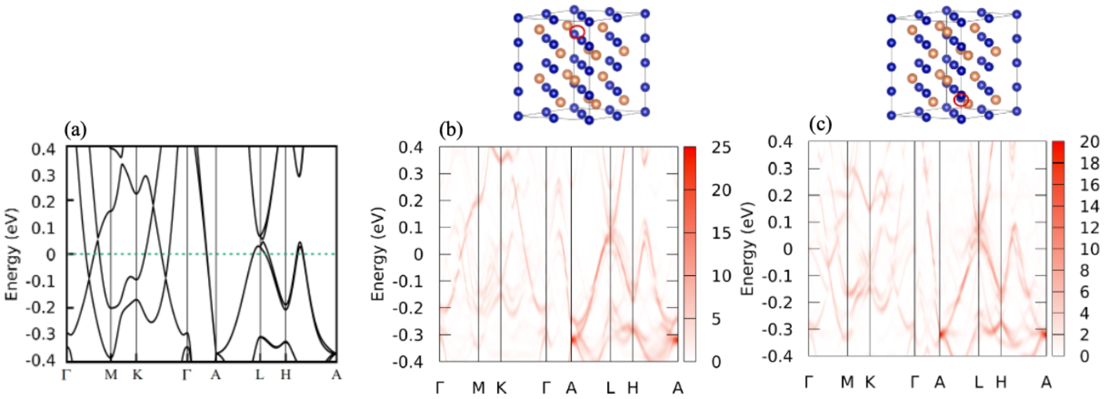

Figure S3. Defects induced band structure changing. (a) Band structure of pure CrSb in the pristine BZ. (b) Band structure of CrSb with Cr vacancies unfolded in the pristine BZ. The upper panel shows the new enlarged unit cell with the red cite as vacancy. (c) Band structure of CrSb with excess Sb unfolded in the pristine BZ. The upper panel shows the new enlarged unit cell with the red cite Cr replaced with Sb. The general band structure keeps same as the pure CrSb.

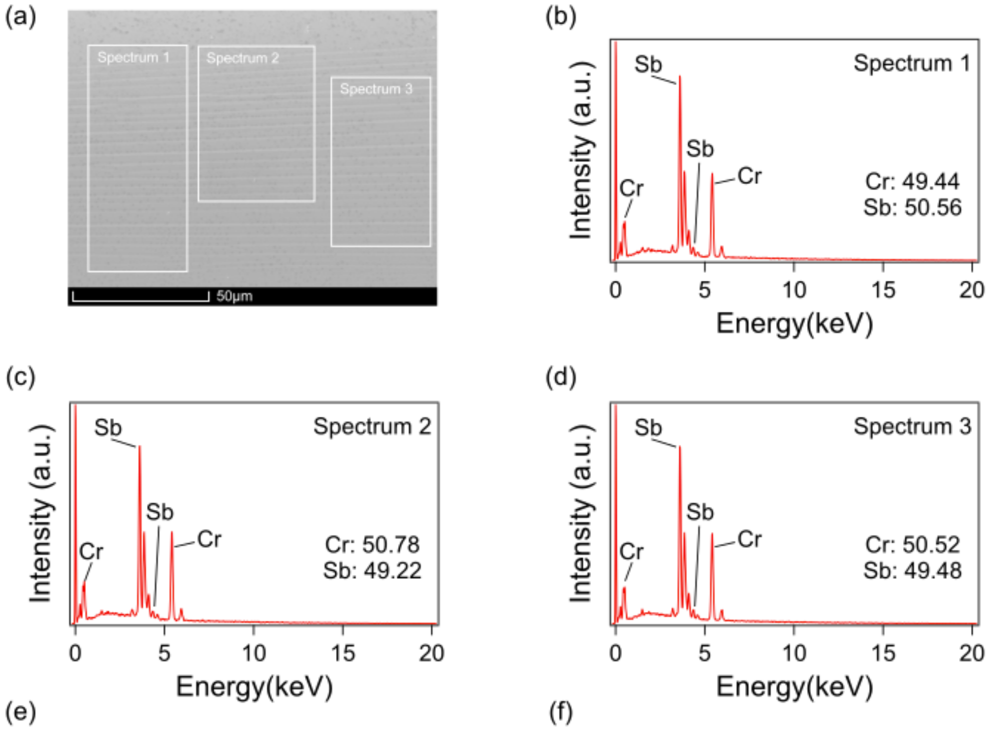

(e)

Label	Cr	Sb	Total
Spectrum 1	49.44	50.56	100.00
Spectrum 2	50.78	49.22	100.00
Spectrum 3	50.52	49.48	100.00

(f)

Statistic	Cr	Sb
Max	50.78	50.56
Min	49.44	49.22
Average	50.25	49.75

Figure S4. EDS results showing the proportion of each element in the sample. (a) Electron image of the sample surface with a magnification of 1000 times, showing the religion where the spectrum is measured. (b-d) Spectrum results in the three religions. (e-f) Detailed proportion of each element of the CrSb sample in the measured religion, indicating the samples do not differ from their stoichiometric phase.

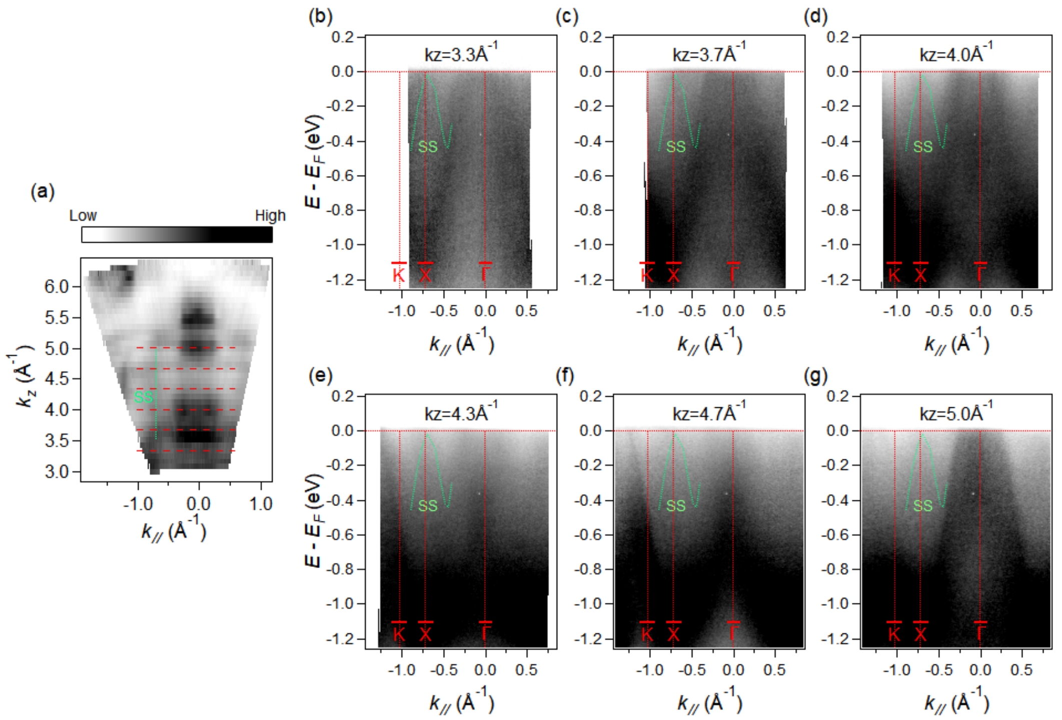

Figure S5. Surface states identified in photon energy-dependent ARPES measurements, exhibiting no k_z dispersion. (a) Fermi surfaces acquired in the plane of k_z-k_x. The red dotted lines indicate the positions of the cuts in panels (b-g). The green dotted line shows the position of the surface states along k_z direction. (b-g) Experimental band spectra along cuts with different k_z marked with red dotted lines in (a). The green dotted lines illustrate the same dispersion of the surface bands across different k_z values.

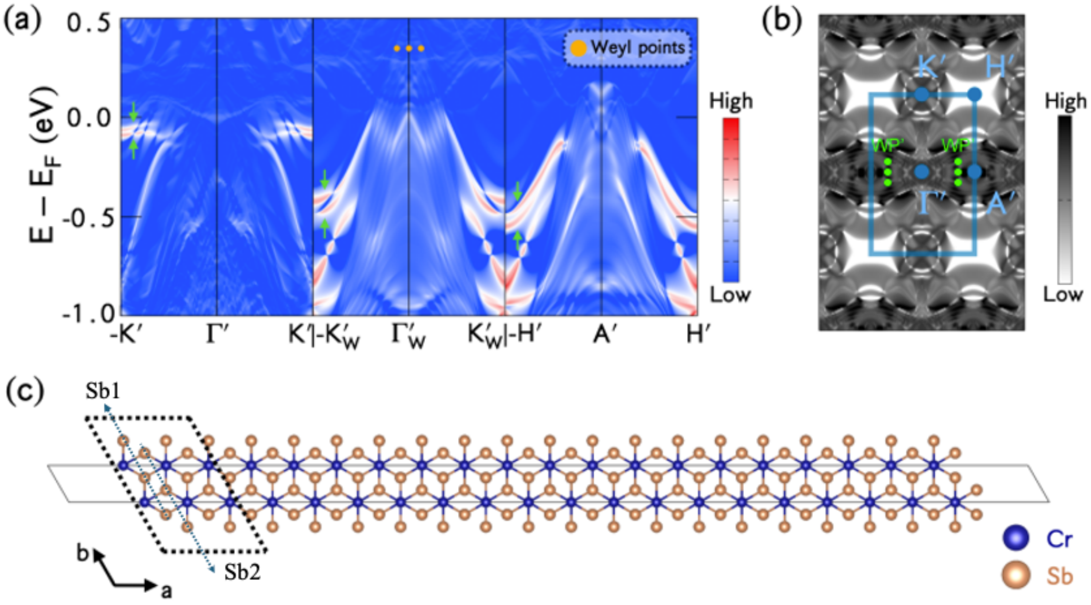

Figure S6. Calculated surface state bands. (a) The calculated (100) surface state along the path $-\mathbf{K}'$-$\mathbf{\Gamma}'$-$\mathbf{K}'|-\mathbf{K}'_{\mathbf{W}}$-$\mathbf{\Gamma}'_{\mathbf{W}}$-$\mathbf{K}'_{\mathbf{W}}|-\mathbf{H}'$-$\mathbf{A}'$-$\mathbf{H}'$ shown in Figure 4(g). The green arrows point to the surface bands. (b) The Fermi surface of (100), with prime notations representing points in the Brillouin zone of (c). (c) Based on the primitive cell, the structure is expanded by a factor of 20 along the (100) direction with Cr atoms exposed at the surface and the regions enclosed by dashed lines represent the areas projected for our calculations of edge states and the Fermi surface.

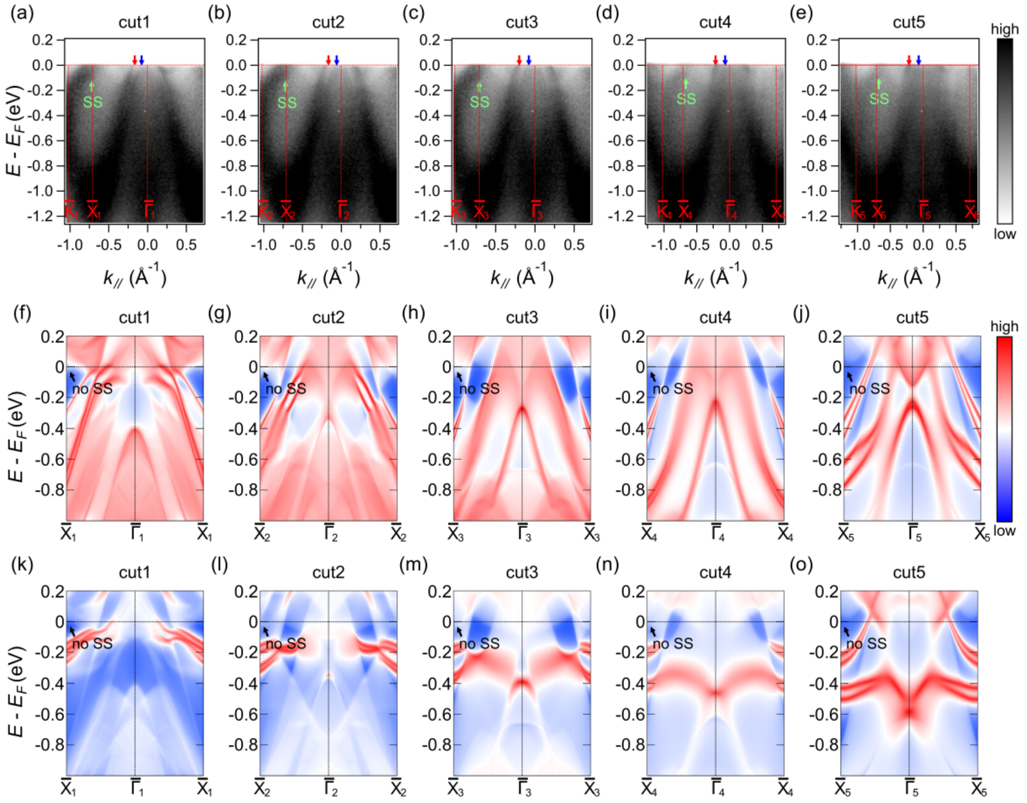

Figure S7. Comparison of experimental results with calculated surface states with two-type Sb terminations. (a-e) Experimental band dispersion along cut1-5, which are the same as shown in Fig. 3 of main text. (f-j) Calculated surface states with Sb-1 termination, as shown in Fig. S2. (k-o) Calculated surface states with Sb-2 termination, as shown in Fig. S2.

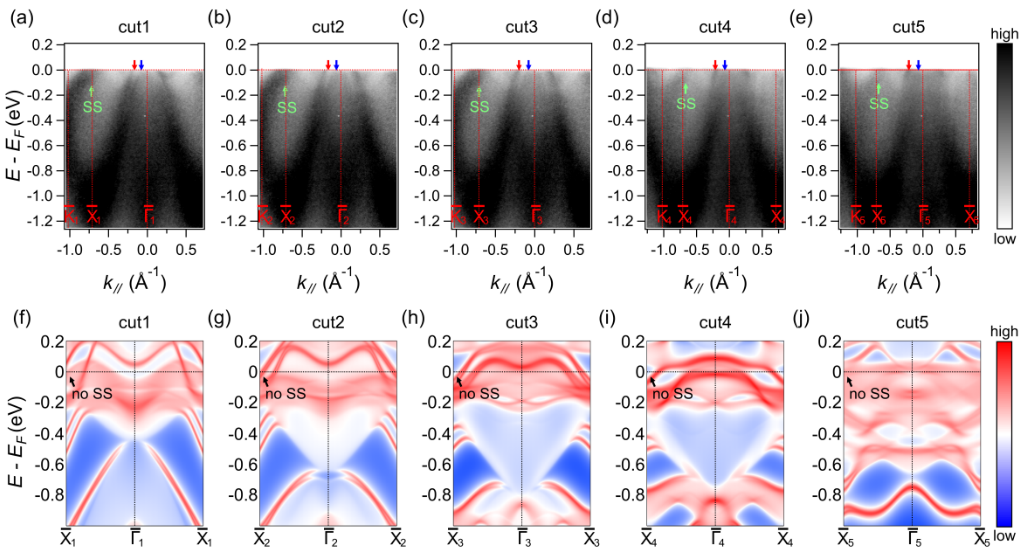

Figure S8. Comparison of experimental results with calculated surface projected states along cut1-5 in the paramagnetic phase. (a-e) Experimental band dispersion along cut1-5, which are the same as shown in Fig. 3 in the main text. (f-j) Calculated surface projected states at the paramagnetic phase along cut1-5 reveal distinct surface states compared to the altermagnetic phase. These states also differ from the experimental results, suggesting that the surface states observed in the experiments are topological nontrivial driven from the altermagntic Weyl physics.

References

(1) Šmejkal, L.; Sinova, J.; Jungwirth, T. Emerging Research Landscape of Altermagnetism. *Phys Rev X* **2022**, *12* (4), 040501. https://doi.org/10.1103/PhysRevX.12.040501.

(2) Šmejkal, L.; Sinova, J.; Jungwirth, T. Beyond Conventional Ferromagnetism and Antiferromagnetism: A Phase with Nonrelativistic Spin and Crystal Rotation Symmetry. *Phys Rev X* **2022**, *12* (3), 031042. https://doi.org/10.1103/PhysRevX.12.031042.

(3) Mazin, I. I. Altermagnetism in MnTe: Origin, Predicted Manifestations, and Routes to Detwinning. *Phys Rev B* **2023**, *107* (10), L100418. https://doi.org/10.1103/PhysRevB.107.L100418.

(4) Cheong, S.-W.; Huang, F.-T. Altermagnetism with Non-Collinear Spins. *NPJ Quantum Mater* **2024**, *9* (1), 13. https://doi.org/10.1038/s41535-024-00626-6.

(5) Naka, M.; Hayami, S.; Kusunose, H.; Yanagi, Y.; Motome, Y.; Seo, H. Spin Current Generation in Organic Antiferromagnets. *Nat Commun* **2019**, *10* (1), 4305. https://doi.org/10.1038/s41467-019-12229-y.

(6) Yuan, L.-D.; Wang, Z.; Luo, J.-W.; Rashba, E. I.; Zunger, A. Giant Momentum-Dependent Spin Splitting in Centrosymmetric Low-*Z* Antiferromagnets. *Phys Rev B* **2020**, *102* (1), 014422. https://doi.org/10.1103/PhysRevB.102.014422.

(7) Šmejkal, L.; González-Hernández, R.; Jungwirth, T.; Sinova, J. Crystal Time-Reversal Symmetry Breaking and Spontaneous Hall Effect in Collinear Antiferromagnets. *Sci Adv* **2020**, *6* (23), eaaz8809. https://doi.org/10.1126/sciadv.aaz8809.

(8) Mazin, I. I.; Koepernik, K.; Johannes, M. D.; González-Hernández, R.; Šmejkal, L. Prediction of Unconventional Magnetism in Doped FeSb2. *Proceedings of the National Academy of Sciences* **2021**, *118* (42) , e2108924118. https://doi.org/10.1073/pnas.2108924118.

(9) González-Hernández, R.; Šmejkal, L.; Výborný, K.; Yahagi, Y.; Sinova, J.; Jungwirth, T.; Železný, J. Efficient Electrical Spin Splitter Based on Nonrelativistic Collinear Antiferromagnetism. *Phys Rev Lett* **2021**, *126* (12), 127701. https://doi.org/10.1103/PhysRevLett.126.127701.

(10) Shao, D.-F.; Zhang, S.-H.; Li, M.; Eom, C.-B.; Tsymbal, E. Y. Spin-Neutral Currents for Spintronics. *Nat Commun* **2021**, *12* (1), 7061. https://doi.org/10.1038/s41467-021-26915-3.

(11) Ma, H.-Y.; Hu, M.; Li, N.; Liu, J.; Yao, W.; Jia, J.-F.; Liu, J. Multifunctional Antiferromagnetic Materials with Giant Piezomagnetism and Noncollinear Spin Current. *Nat Commun* **2021**, *12* (1), 2846. https://doi.org/10.1038/s41467-021-23127-7.

(12) Šmejkal, L.; MacDonald, A. H.; Sinova, J.; Nakatsuji, S.; Jungwirth, T. Anomalous Hall Antiferromagnets. *Nat Rev Mater* **2022**, *7* (6), 482–496. https://doi.org/10.1038/s41578-022-00430-3.

(13) Fernandes, R. M.; de Carvalho, V. S.; Birol, T.; Pereira, R. G. Topological Transition from Nodal to Nodeless Zeeman Splitting in Altermagnets. *Phys Rev B* **2024**, *109* (2), 024404. https://doi.org/10.1103/PhysRevB.109.024404.

(14) Bose, A.; Schreiber, N. J.; Jain, R.; Shao, D.-F.; Nair, H. P.; Sun, J.; Zhang, X. S.; Muller, D. A.; Tsymbal, E. Y.; Schlom, D. G.; Ralph, D. C. Tilted Spin Current Generated by the Collinear Antiferromagnet Ruthenium Dioxide. *Nat Electron* **2022**, *5* (5), 267–274. https://doi.org/10.1038/s41928-022-00744-8.

(15) Šmejkal, L.; Hellenes, A. B.; González-Hernández, R.; Sinova, J.; Jungwirth, T. Giant and Tunneling Magnetoresistance in Unconventional Collinear Antiferromagnets with Nonrelativistic Spin-Momentum Coupling. *Phys Rev X* **2022**, *12* (1), 011028. https://doi.org/10.1103/PhysRevX.12.011028.

(16) Karube, S.; Tanaka, T.; Sugawara, D.; Kadoguchi, N.; Kohda, M.; Nitta, J. Observation of Spin-Splitter Torque in Collinear Antiferromagnetic RuO2. *Phys Rev Lett* **2022**, *129* (13), 137201. https://doi.org/10.1103/PhysRevLett.129.137201.

(17) Zhou, X.; Feng, W.; Yang, X.; Guo, G.-Y.; Yao, Y. Crystal Chirality Magneto-Optical Effects in Collinear Antiferromagnets. *Phys Rev B* **2021**, *104* (2), 024401. https://doi.org/10.1103/PhysRevB.104.024401.

(18) Naka, M.; Hayami, S.; Kusunose, H.; Yanagi, Y.; Motome, Y.; Seo, H. AnAnomalous Hall Effect in κ-Type Organic Antiferromagnets. *Phys Rev B* **2020**, *102* (7), 075112. https://doi.org/10.1103/PhysRevB.102.075112.
